# Novel Insight Into Glycosaminoglycan Biosynthesis Based on Gene Expression Profiles

**DOI:** 10.3389/fcell.2021.709018

**Published:** 2021-09-06

**Authors:** Yi-Fan Huang, Shuji Mizumoto, Morihisa Fujita

**Affiliations:** ^1^Key Laboratory of Carbohydrate Chemistry and Biotechnology, Ministry of Education, School of Biotechnology, Jiangnan University, Wuxi, China; ^2^Department of Pathobiochemistry, Faculty of Pharmacy, Meijo University, Nagoya, Japan

**Keywords:** dermatan sulfate (DS), chondroitin sulfate (CS), glycosaminoglycan (GAG), GlycoMaple, heparan sulfate (HS), hyaluronan (HA), keratan sulfate (KS), proteoglycan (PG)

## Abstract

Glycosaminoglycans (GAGs) including chondroitin sulfate, dermatan sulfate, heparan sulfate, and keratan sulfate, except for hyaluronan that is a free polysaccharide, are covalently attached to core proteins to form proteoglycans. More than 50 gene products are involved in the biosynthesis of GAGs. We recently developed a comprehensive glycosylation mapping tool, GlycoMaple, for visualization and estimation of glycan structures based on gene expression profiles. Using this tool, the expression levels of GAG biosynthetic genes were analyzed in various human tissues as well as tumor tissues. In brain and pancreatic tumors, the pathways for biosynthesis of chondroitin and dermatan sulfate were predicted to be upregulated. In breast cancerous tissues, the pathways for biosynthesis of chondroitin and dermatan sulfate were predicted to be up- and down-regulated, respectively, which are consistent with biochemical findings published in the literature. In addition, the expression levels of the chondroitin sulfate-proteoglycan versican and the dermatan sulfate-proteoglycan decorin were up- and down-regulated, respectively. These findings may provide new insight into GAG profiles in various human diseases including cancerous tumors as well as neurodegenerative disease using GlycoMaple analysis.

## Introduction

Glycosaminoglycans (GAGs) are linear polysaccharides consisting of repeating disaccharide units. Among these are heparin, heparan sulfate (HS), chondroitin sulfate (CS), dermatan sulfate (DS), and keratan sulfate (KS), which are covalently bound to core proteins, forming proteoglycans (PGs) (Kjellen and Lindahl, 1991; Iozzo, 1998; Lindahl et al., 2015). Hyaluronan (HA) is a free polysaccharide (Lindahl and Rodén, 1972; Hascall, 2019). PGs are distributed in the extracellular matrix and on cell surfaces, and are crucially involved in a wide range of biological processes such as cell adhesion, the regulation of cellular signaling, and assembly of the extracellular matrix (Esko and Selleck, 2002; Häcker et al., 2005; Bülow and Hobert, 2006; Bishop et al., 2007; Sarrazin et al., 2011; Thelin et al., 2013; Xu and Esko, 2014; Mizumoto et al., 2015; Neill et al., 2015).

CS, DS, and HS are covalently linked to specific serine residues usually flanked by a glycine residue on core proteins via a common linker tetrasaccharide region, GlcAβ1–3Gaβ1–3Galβ1–4Xylβ1–*O*–, where GlcA, Gal, and Xyl stand for glucuronic acid, galactose, and xylose, respectively (Lindahl and Rodén, 1972; Sugahara and Kitagawa, 2000). The biosynthesis of the linker tetrasaccharide is initiated by the transfer of β-Xyl from uridine diphosphate (UDP)-Xyl to the specific serine residue(s) on the core proteins of PGs by β-xylosyltransferase (XYLT) encoded by *XYLT1* or *XYLT2* (Götting et al., 2000; Pönighaus et al., 2007) at the endoplasmic reticulum, endoplasmic reticulum-Golgi intermediate compartment, or *cis*-Golgi apparatus (Prydz, 2015). Then, a second Gal is transferred from UDP-Gal to Xylβ-*O*-serine by β4-galactosyltransferase-I encoded by *B4GALT7* (Almeida et al., 1999; Okajima et al., 1999). The C2-position of Xyl is phosphorylated by xylosylkinase encoded by *FAM20B* (Koike et al., 2009). A third Gal is added to the second Gal residue on the Galβ1–4Xylβ-*O*-serine from UDP-Gal by β3-galactosyltransferase-II encoded by *B3GALT6* (Bai et al., 2001). β3-Glucuronosyltransferase-I encoded by *B3GAT3* transfers GlcA to Galβ1–3Galβ1–4Xylβ-*O*-serine from UDP-GlcA (Kitagawa et al., 1998). Then, a phosphate group in GlcAβ1–3Galβ1–3Galβ1–4Xyl(2-*O*-phosphate) is removed by 2-*O*-phosphoxylose phosphatase encoded by *PXYLP1* (Koike et al., 2014). The types of GAGs including CS, DS, or HS extended from the linker tetrasaccharide are determined by the structure of the core protein, sulfation and phosphorylation status on the tetrasaccharide, and the biochemical environment of GAGosome, which is a complex of enzymes and regulatory factors in the Golgi apparatus (Sugahara and Kitagawa, 2000; Presto et al., 2008; Prydz, 2015; Izumikawa, 2019).

Heparan sulfate consists of repeating disaccharide units of *N*-acetylglucosamine (GlcNAc) (Rodén et al., 1992), and GlcA that are polymerized onto the linker tetrasaccharide region of specific core proteins (Lidholt et al., 1989; Lindahl, 1989; Esko and Selleck, 2002; Kim et al., 2003). The initial GlcNAc residue is transferred from UDP-GlcNAc to the tetrasaccharide by α4-*N*-acetylglucosaminyltransferase (GlcNAcT)-I encoded by *exostosin-like 2* (*EXTL2*) or *EXTL3* (Kitagawa et al., 1999; Kim et al., 2001). Thereafter, polymerization of the HS-repeating disaccharide region, [–3GalNAcα1–4GlcAβ1–]_*n*_, occurs by enzymatic activities designated as HS-β4-glucuronosyltransferase-II (HS-GlcAT-II) and GlcNAcT-II, which are catalyzed by a HS polymerase enzyme hetero-complex composed of exostosin 1 (EXT1) and EXT2 (Lind et al., 1998; McCormick et al., 1998; Kim et al., 2003). EXTL1 and EXTL3 also show GlcNAcT-II activity (Kim et al., 2001). The GlcNAc residue on the repeating unit is partially converted to *N*-sulfated glucosamine by a dual enzyme, *N*-deacetylase/*N*-sulfotransferase encoded by *NDST1*, *NDST2*, *NDST3*, or *NDST4* (Hashimoto et al., 1992; Eriksson et al., 1994; Aikawa and Esko, 1999; Aikawa et al., 2001). HS C5-epimerase encoded by *GLCE* converts GlcA residues, which are located on the non-reducing side of *N*-sulfated glucosamine in the repeating disaccharide region of HS, iduronic acid (IdoA) (Li et al., 1997). The disaccharide region can be further *O*-sulfated at the C2 position of IdoA by HS 2-*O*-sulfotransferases, and *O*-sulfated at C3 and C6 positions of GlcNAc or *N*-sulfated glucosamine by HS 3-*O*-sulfotransferases and HS 6-*O*-sulfotransferases, respectively. 3′-phosphoadenosine 5′-phosphosulfate (PAPS) is utilized as the substrate for the sulfation reaction (Kusche-Gullberg and Kjellen, 2003). PAPS is synthesized from ATP and an inorganic sulfate in the cytosol by PAPS synthase encoded by *PAPSS1* or *PAPSS2* (Venkatachalam, 2003). PAPS is transported into the Golgi lumen from the cytosol by two PAPS transporters encoded by *SLC35B2* and *SLC35B3* (Kamiyama et al., 2003, 2006).

The repeating disaccharide region of CS and DS, [–4GlcAβ1–3*N*-acetylgalactosamine (GalNAc)β1–]_*n*_ and [–4IdoAβ1–3GalNAcβ1–]_*n*_, respectively, is also attached to a linker tetrasaccharide, GlcA-Gal-Gal-Xyl, on a serine residue of specific core proteins (Lindahl and Rodén, 1972; Fransson et al., 1993; Lamari and Karamanos, 2006). The initial GalNAc residue is transferred from UDP-GalNAc to the tetrasaccharide by β4-*N*-acetylgalactosaminyltransferase (GalNAcT)-I encoded by *CSGALNACT1* or *CSGALNACT*2 (Uyama et al., 2002, 2003). The CS-repeating disaccharide region is formed by the alternative addition of GlcA and GalNAc residues from UDP-GlcA and UDP-GalNAc to the non-reducing end of the linker region tetrasaccharide, GlcA-Gal-Gal-Xyl, by CS-GlcAT-II and GalNAcT-II activities, respectively, of a chondroitin synthase (CHSY) family member including CHSY1, CHSY3, chondroitin polymerizing factor (CHPF), and CHPF2 (Kitagawa et al., 2001, 2003; Izumikawa et al., 2007, 2008). The combination of any two heterocomplexes of these five proteins exerts polymerization activity to build the repeating disaccharide region of CS (Kitagawa et al., 2001, 2003; Izumikawa et al., 2007, 2008). After or during biosynthesis of the disaccharide region, GlcA residues in the chondroitin precursor chain are epimerized to IdoA by DS-epimerase encoded by *DSE* or *DSEL* (Maccarana et al., 2006; Pacheco et al., 2009). The CS and DS repeating disaccharide regions, [–4GlcAβ1–3GalNAcβ1–]_*n*_ and [–4IdoAβ1–3GalNAcβ1–]_*n*_, respectively, can be modified by sulfation at C4 and C6 positions of GalNAc and C2 position of GlcA and IdoA by chondroitin 4-*O*-sulfotransferases, chondroitin 6-*O*-sulfotransferases, and uronyl 2-*O*-sulfotransferase, respectively (Kusche-Gullberg and Kjellen, 2003).

Keratan sulfate consists of sulfated poly-*N*-acetyllactosamine, [–4GlcNAcβ1–3Galβ1–]_*n*_, which is bound to serine, threonine, or asparagine on specific core proteins through the linkage region such as O-linked or N-linked oligosaccharides, respectively (Stuhlsatz et al., 1989; Caterson and Melrose, 2018). The biosynthesis of the repeating disaccharide region of KS is initiated by β3-*N*-acetylglucosaminyltransferase encoded by *B3GNT7* (Seko and Yamashita, 2004; Kitayama et al., 2007). Thereafter, GlcNAc 6-*O*-sulfotransferase encoded by *CHST5* or *CHST6* transfers a sulfate group from PAPS to the GlcNAc residue (Akama et al., 2001, 2002; Narentuya et al., 2019), following the addition of a Gal residue to the GlcNAc6-*O*-sulfate residue by β4-galactosyltransferase encoded by *B4GALT4* (Seko et al., 2003; Kitayama et al., 2007). After the construction of polysaccharide, [–4GlcNAc(6S)β1–3Galβ1–]_*n*_, KS Gal 6-*O*-sulfotransferase is encoded by *CHST1* (Fukuta et al., 1997; Akama et al., 2002). KS-PGs are distributed widely in the cornea, cartilage, and brain, and play important roles in collagen fibrillogenesis, tissue hydration, neurotransmission, and nerve regeneration (Caterson and Melrose, 2018).

Hyaluronan is a high molecular weight, natural polymer composed of a repeating disaccharide, [–4GlcAβ1–3GlcNAcβ1–]_*n*_ (Rodén, 1980; Hascall and Esko, 2015; Abbruzzese et al., 2017; Hascall, 2019). HA is synthesized from cytosolic UDP-GlcNAc and UDP-GlcA by hyaluronan synthase 1 (HAS1), HAS2, or HAS3 at the plasma membrane (Itano and Kimata, 2002), whereas other GAGs are synthesized in the secretory pathway. HAS1 and HAS2 contribute to the synthesis of longer hyaluronan (∼2 × 10^6^ Da and >2 × 10^6^ Da, respectively), whereas HAS3 synthesizes a markedly shorter hyaluronan (∼1 × 10^6^ Da) (Itano et al., 1999). HA is a major component of the extracellular matrix, and is not covalently attached to a core protein (Lindahl and Rodén, 1972; Hascall, 2019). During the tissue injury and repair process, HA is actively produced and plays important roles (Liang et al., 2016).

The abundance of GAGs and their pattern of sulfation are dynamically altered, affecting GAG–protein interactions (Kjellen and Lindahl, 2018) during a number of physiological and pathological processes, such as differentiation, tumorigenesis, inflammation, and bacterial and viral infections (Sasisekharan et al., 2002; Bishop et al., 2007; Iozzo and Sanderson, 2011; Jiang et al., 2011; Kam and Alexander, 2014; Baghy et al., 2016; Morla, 2019). Therefore, the analyses of changes in content as well as sulfation pattern of GAGs are required to elucidate the pathogenesis. However, these analyses are complicated and require special techniques. We previously established GlycoMaple, a comprehensive glycosylation mapping tool based on genetic expression profiles (Huang et al., 2021). With this tool, the expression profiles of glycan-related genes are mapped into glycan metabolic pathways to visualize and estimate glycan biosynthesis or degradation using transcriptional data. Transcripts per million transcripts (TPM) values of genes in RNA-Seq analyses are commonly utilized to evaluate and compare gene expression levels among samples from distinct cells or tissues. By uploading RNA-Seq data, glycan metabolic pathways in the tissue of interest can be automatically drawn to understand the glycosylation process. In this study, the expression levels of genes involved in GAG biosynthetic pathways in various tissues and tumor tissues have been addressed using gene expression profile data available in public databases, thereby allowing the estimation and comparison of amounts as well as sulfation pattern of GAGs between normal and disease states.

## Methods

Expression data of GAG-related genes (Supplementary Table 1) in 37 human tissues were downloaded from the Human Protein Atlas database^1^ (Supplementary Table 2). TPM values of GAG-related genes (Supplementary Table 1) and PG genes (Supplementary Table 3) from healthy tissue in GTEx and primary tumor tissues in TCGA were obtained from UCSC Xena^2^ (Goldman et al., 2020). Raw data (log_2_[TPM + 0.001]) in Xena were converted to TPM values (Supplementary Tables 4, 6). TPM values of samples were input in GlycoMaple, and then pathways were visualized (Huang et al., 2021). When several genes overlapped in a reaction, the maximum TPM value among the values of overlapping genes was used to represent this reaction. When several gene products comprised a reaction complex, the minimum TPM value of the subunit genes was used to represent the reaction. To compare GAG biosynthetic pathways between tumor and normal tissues, fold changes in expression of genes, whose median TPM + 1 values increased by >1.5 and decreased by <0.667, are shown as pink and green arrows, respectively, in the pathways. The heatmaps and boxplots for gene expression profiles were drawn by R (ver. 3.6.2) (R Foundation for Statistical Computing, Vienna, Austria). The data used for boxplots were converted to log_2_ (TPM + 1). The Wilcoxon matched-pairs signed rank test was used to compare gene expression levels between primary tumor and normal tissues.

## Results and Discussion

### GAG Expression Profiles of Human Tissues

We previously listed 950 human glycan-related genes and established a tool named GlycoMaple, which can visualize 19 human glycan metabolic pathways and estimate the glycan structures synthesized in cells or tissues (Huang et al., 2021). In this study, we focused on GAG biosynthetic pathways. Among 950 glycan-related genes, those involved in: (1) biosynthesis of GAG backbones, (2) sulfation modification of HS, (3) sulfation modification of CS and DS, (4) biosynthesis of KS, and (5) biosynthesis and catabolism of HA in GlycoMaple pathways, were selected as GAG-related genes (Supplementary Figure 1). In addition, genes encoding PAPS transporters (*SLC35B2* and *SLC35B3*), a UDP-Xyl and UDP-GlcNAc transporter (*SLC35B4*), and a UDP-Gal transporter (*SLC35A2*) were included. *PAPSS1* and *PAPSS2*, *CANT1*, and *IMPAD1/BPNT2* encoding PAPS synthases 1 and 2, calcium-activated nucleotidase 1/UDP-diphosphatase, and 3′-phosphoadenosine 5′-phosphate 3′-phosphatase, respectively, were also listed. Furthermore, genes encoding regulators of GAG biosynthesis such as maintenance of Golgi-resident glycosyltransferases (*GOLPH3*) (Chang et al., 2013), a Golgi-localized protease (*SPPL3*) (Kuhn et al., 2015), a transcriptional regulator (*ZNF263*) (Weiss et al., 2020), and an epigenetic factor (*KDM2B*) (Weiss et al., 2021) were also added to the list. In total, 66 genes were listed in these categories (Supplementary Table 1).

**FIGURE 1:**
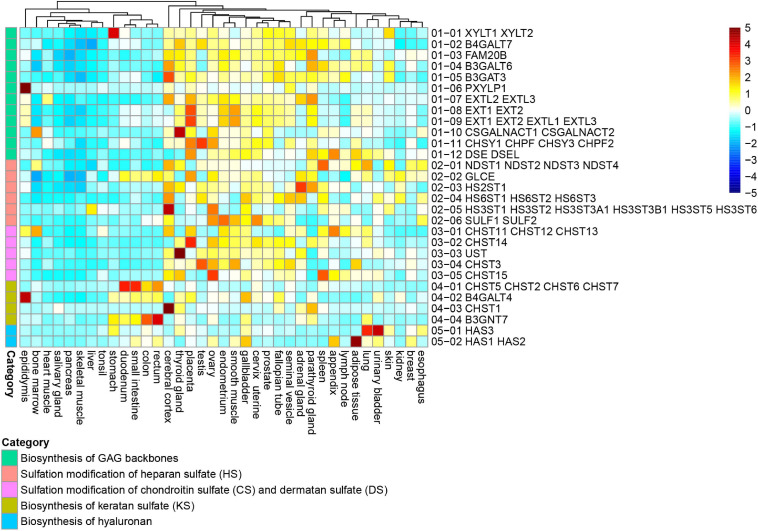
Expression profiles of GAG-related genes in human tissues. Expression data of genes responsible for 29 reactions in five categories including biosynthesis of GAG backbones (green), sulfation of HS (red), sulfation of CS and DS (pink), biosynthesis of KS (ocher), and biosynthesis of HA (blue). Expression data were obtained from 37 human tissues of the Human Protein Atlas. The reaction numbers are correlated with Supplementary Figure 1 and Supplementary Table 1. Transcripts per million transcripts (TPM) values in each of 29 reactions were normalized (*z*-score) and visualized as a heatmap.

To evaluate GAG biosynthesis capability in human tissue, we used RNA-Seq data from 37 human tissues, obtained from the Human Protein Atlas (Pontén et al., 2008). TPM is a value that indicates how many transcripts are present in each transcript when there are 1 million total transcripts in the sample (Wagner et al., 2012). The TPM is a method frequently utilized for comparing samples. First, gene expression profiles in each reaction were compared among tissues (Figure 1). The tissues were clustered into two groups by gene expression profiles of GAG biosynthetic reactions. One cluster included the cerebral cortex, thyroid gland, placenta, testis, ovary, endometrium, smooth muscle, cervix/uterine, prostate, fallopian tube, seminal vesicle, adrenal gland, parathyroid gland, spleen, lymph node, skin, lung, urinary bladder, appendix, and gallbladder. This cluster showed relatively higher expression of GAG-related genes, suggesting stronger productive ability of GAGs. In contrast, tissues such as the tonsil, liver, skeletal muscle, pancreas, salivary gland, and heart muscle were clustered together in the same tree because of lower expression of GAG-related genes. Certain digestive organs including the duodenum, small intestine, colon, and rectum were sub-clustered in this group because of the relatively low expression of genes involved in the biosynthesis of HS, CS, and DS. Instead, genes required for KS biosynthesis were expressed in the digestive tissues.

**FIGURE 2:**
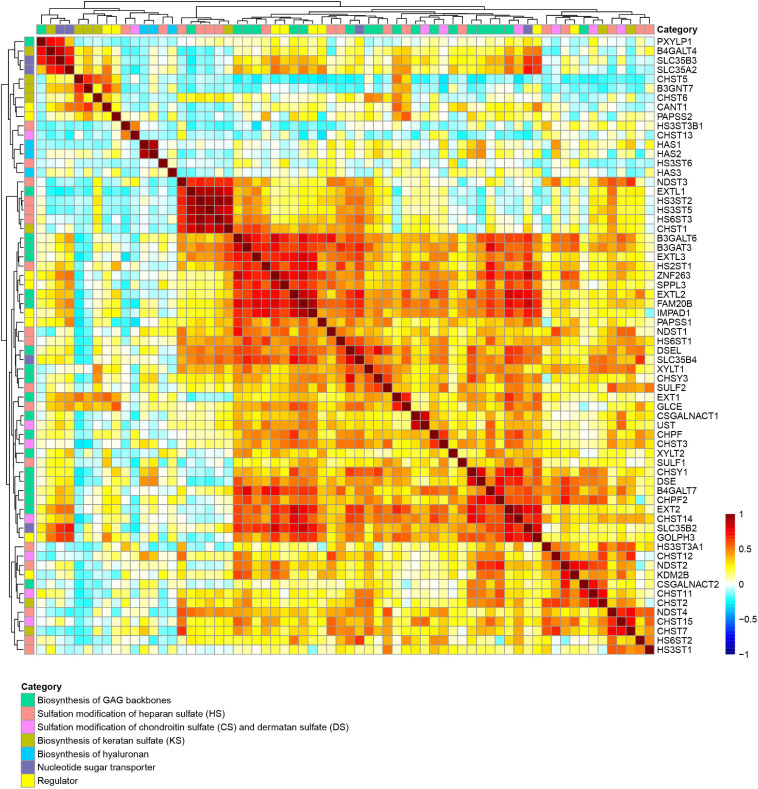
Correlation of expression of GAG-related genes among human tissues. Similarity of expression patterns of 56 genes involved in GAG biosynthesis in 37 human tissues, including biosynthesis of GAG backbones (green), sulfation of HS (red), sulfation of CS and DS (pink), biosynthesis of KS (ocher), biosynthesis of HA (blue), nucleotide sugar transporters (purple), and regulators (yellow) were visualized as a Pearson correlation heatmap. The Pearson correlation values (–1 to 1) are presented as color changes (blue to red).

We next examined the Pearson correlation of 66 GAG-related gene expression in 37 human tissues to identify similarities in the expression patterns. In the Pearson correlation heatmap for GAG-related genes, many genes required for the biosynthesis of GAG backbones, including *XYLT1*, *XYLT2*, *FAM20B*, *B4GALT7*, *B3GALT6*, *B3GAT3*, *EXT1*, *EXT2*, *EXTL2*, *EXTL3*, *CHSY1*, *CHSY3*, *CHPF*, *CHPF2*, *CSGALNACT1*, *DSE*, and *DSEL*, clustered together (Figure 2 and Supplementary Table 2). The expression patterns of regulator genes for GAG biosynthesis including *SPPL3*, *GOLPH3*, and *ZNF263*, and *SLC35B2*, *SLC35B4*, *PAPSS1*, and *IMPAD1*, which encode a PAPS transporter, UDP-Xyl/UDP-GlcNAc dual transporter, PAPS synthase 1, and 3′-phosphoadenosine 5′-phosphate 3′-phosphatase, respectively, were also correlated with genes required for biosynthesis of sulfated GAGs in human tissues (Figure 2 and Supplementary Table 2). These findings suggest that expression levels of these genes are regulated by similar mechanisms such as regulation by corresponding signal transductions as well as transcriptional factors. On the other hand, some other genes encoding regulators and transporters were not clustered in the group (Figure 2). For example, the expression patterns of *SLC35A2*, whose gene product imports UDP-Gal into the lumen of the Golgi apparatus, were different from those of genes categorized as necessary for the biosynthesis of GAG backbones. This is reasonable because UDP-Gal is utilized not only for GAG biosynthesis, but also for biosynthesis of other glycans, such as *N*-glycans, mucin-type *O*-glycans, and glycolipid. *HAS1* and *HAS2*, which encode hyaluronan synthase to produce a higher molecular weight form of HA, shared close expression patterns (Figure 2), indicating linkage between gene function and transcriptional regulation. Genes required for sulfation of GAGs were distributed in different trees, although some of them, such as *NDST3*, *HS3ST2*, *HS3ST3*, and *HS3ST5*, showed correlated expression patterns in normal human tissues (Figure 2 and Supplementary Table 2). It has been reported that several genes such as *B3GAT3*, *EXT2*, *GLCE*, *NDST2*, *HS3ST1*, *HS3ST6*, *HS6ST1*, and *CSGALNACT2* are upregulated by treatment with 4-methylumbelliferyl-β-xyloside, which inhibits the biosynthesis of GAGs (Sasaki et al., 2019). Some of these genes possess enhancer elements named PG stress response elements, PGSE-A and PGSE-B, in their promoter region, which regulate the transcription activation upon Golgi stress caused by PG defects (Sasaki et al., 2019). Among these genes, *CSGALNACT2*, *NDST2*, and *HS3ST1* were clustered in the Pearson correlation heatmap (Figure 2), suggesting that their expression levels are regulated in a common manner.

### Prediction of Alterations in GAG Structure in Human Tumor Tissues

Because GAGs are involved in cell adhesion, migration, proliferation, and inflammation, changes in GAG levels can affect tumor development (Sasisekharan et al., 2002; Bishop et al., 2007; Iozzo and Sanderson, 2011; Mizumoto and Sugahara, 2013; Wei et al., 2020). The GlycoMaple tool can compare glycan metabolic pathways between two samples based on gene expression data. The comparison function enables us to reveal underlying changes of PGs or GAGs between normal and tumor tissues. We compared GAG biosynthetic pathways between several primary tumor and normal tissues using RNA-Seq data from two public databases: the Cancer Genome Atlas Program (TCGA) and Genotype-Tissue Expression Project (GTEx) (GTEx Consortium, 2017; Hoadley et al., 2018). TPM values of GAG-related genes were used as expression values, which were obtained from RNA-Seq data of primary tumor and normal tissue samples (brain, pancreas, breast, adrenal glands, and thyroid glands) processed in UCSC Xena (Goldman et al., 2020). Based on these expression values, GAG biosynthetic pathways were then analyzed using GlycoMaple.

**FIGURE 3:**
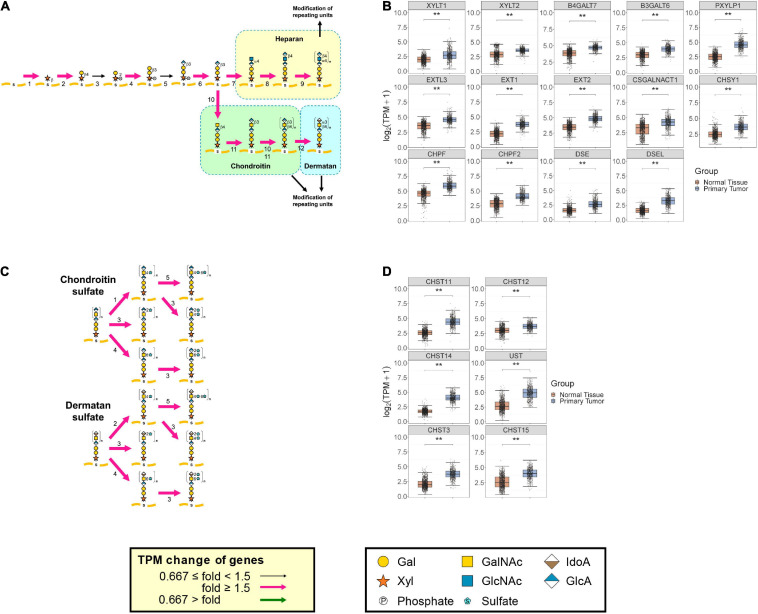
Comparison of expression of GAG biosynthetic enzymes between primary brain tumors and normal tissues. Expression changes of genes responsible for reactions in pathways for biosynthesis of GAG backbones **(A)** and sulfation of CS and DS **(C)** between primary brain tumors (*N* = 662) and normal tissues (*N* = 1146) were visualized using GlycoMaple. Median TPM values of GAG-related genes in each sample were used for mapping. If several genes overlapped in a reaction, the maximum TPM value among the overlapping genes was used. When several gene products comprised a reaction complex, the minimum TPM value of the subunit genes was used. Fold changes of gene expression whose median TPM + 1 value was increased by >1.5 are shown as pink arrows. Genes that are significantly changed among tumor and normal tissues (Wilcoxon matched-pairs signed rank test; **P < 0.0001) in GAG backbones **(B)** and sulfation of CS and DS **(D)** are shown as boxplots. The numbers of each step indicate the enzymes responsible for biosynthesis of GAG backbones **(A)** and sulfation of CS and DS **(B)**. **(A)** 1, *XYLT1* and *XYLT2*; 2, *B4GALT7*; 3, *FAM20B*; 4, *B3GALT6*; 5, *B3GAT3*; 6, *PXYLP1*; 7, *EXTL2* and *EXTL3*; 8 and 9, *EXT1* and *EXT2*; 10, *CSGALNACT1* and *CSGALNACT2*; 11, *CHSY1*, *CHSY3*, *CHPF*, and *CHPF2*; 12, *DSE* and *DSEL*. **(C)** 1, *CHST11*, *CHST12*, and *CHST13*; 2, *CHST14*; 3, *UST*; 4, *CHST3*; 5, *CHST15*.

Spatio/temporal expression of PGs and biosynthesis of GAGs are critical for establishment and maintenance of fundamental functions of the central nervous system (Karamanos et al., 2018). In brain cancers, various GAG and PG levels have been reported to change (Vitale et al., 2019). The medians of TPM from 662 primary brain tumors and 1,146 normal brain tissues were added into the glycosylation pathways. Pink and green arrows indicate whether expression of the genes (TPM + 1) coding the GAG biosynthetic enzymes were increased more than 1.5-fold and decreased to less than 0.667-fold, respectively, in primary tumors, when compared with the corresponding normal tissues (Figure 3). In primary brain tumor samples, many of the reaction steps in GAG biosynthesis were upregulated (Figure 3). In particular, almost all of the reactions involved in the biosynthesis of GAG backbones were markedly increased (Figure 3A). The expression levels of *XYLT1*, *XYLT2*, *B4GALT7*, *B3GALT6*, *PXYLP1*, *EXT1*, *EXT2*, *EXTL3*, *CHSY1*, *CHPF*, *CHPF2*, *CSGALNACT1*, *DSE*, and *DSEL* were increased (Figure 3B). In addition, sulfation of chondroitin and dermatan was predicted to increase in the brain during tumorigenesis, as contributed by *CHST3*, *CHST11*, *CHST12*, *CHST14*, *CHST15*, and *UST* (Figures 3C,D). Besides, the expression levels of several genes encoding core proteins of PG were increased to varying degrees (Supplementary Figure 2A and Supplementary Table 4). It has been reported that various PGs are upregulated in brain cancers. For example, high levels of HA receptors including *epican* (*CD44*) were correlated with poor prognosis in cancer patients (Yan and Wang, 2020). RNA levels for *fibronectin* (*FN1*), *brevican* (*BCAN*), *versican* (*VCAN*), *perlecan* (*HSPG2*), and several laminins were high in glioblastomas compared with in the normal brain (Dzikowski et al., 2021; von Spreckelsen et al., 2021). A high level of *CD74* was expressed in the monocytic subset of immune suppressive myeloid-derived suppressor cells and localized in the tumor microenvironment (Alban et al., 2020). *Glypican 2* (*GPC2*) was selectively expressed in neuroblastoma (Nagarajan et al., 2018). In our analysis, particularly, transcript levels of *CD44*, *BCAN*, *VCAN*, *CD74*, and *GPC2* increased more than five-fold and were the most upregulated PGs in brain tumor tissues, whereas only the expression of *GPC5* was downregulated among the PGs (Supplementary Figure 2A and Supplementary Tables 4, 5). This prediction is consistent with previous reports that PGs help drive multiple oncogenic pathways in tumor cells and promote critical tumor–microenvironment interactions in cancer (Iozzo and Sanderson, 2011; Wade et al., 2013; Theocharis et al., 2015; Schaefer et al., 2017; Yan and Wang, 2020). Furthermore, the expression levels of genes involved in the regulation of GAG biosynthesis such as *SPPL3*, *GOLPH3*, *ZNF263*, and *KDM2B*; in the biosynthesis of a sulfate donor, PAPS synthase encoded by *PAPSS1* and *PAPSS2*; in the transport of PAPS encoded by *SLC35B2*; and in the catabolism of UDP and 3′-phosphoadenosine 5′-phosphate encoded by *CANT1* and *IMPAD1*, respectively, were also increased by more than 1.5 times in brain tumors compared with normal tissues (Supplementary Figure 2B and Supplementary Table 4). These results suggest that demand for the biosynthesis and sulfation of GAGs changes the expression of regulator genes, which might affect the amount as well as sulfation patterns of GAGs. In contrast, the expression levels of biosynthetic enzymes for HA and KS did not show a definite trend between normal and tumor tissues based upon GlycoMaple predictions (data not shown). This indicates that expression levels of biosynthetic enzymes for HA as well as KS and catabolic enzymes for HA might not significantly change, at least in the brain tumors examined.

We also analyzed GAG pathways in pancreatic carcinoma. GAGs are produced at low levels in healthy pancreatic tissue (Theocharis et al., 2000). In contrast, the amounts of GAGs in human pancreatic carcinoma are increased 4-fold, and in particular, show a 22-fold increase of CS and 12-fold increase of HA (Theocharis et al., 2000). The emerging expression of GAGs is considered to be a biological tumor marker for pancreatic tissues. The medians of TPM from 178 primary pancreatic tumors and 165 normal tissues were used to visualize the glycosylation pathways in GlycoMaple. Almost all steps involved in GAG biosynthesis including HS, CS, DS, KS, and HA were expressed in the pancreatic tumor tissues, and increased markedly compared with healthy tissues (Supplementary Figure 3), which was partially consistent with previous reports (Theocharis et al., 2000; Skandalis et al., 2006). It should be noted, however, that the reports showed no changes in the content or molecular size of HS, suggesting that *in silico* analysis is not always correct. In addition, it was reported that 6-*O*-sulfated CS on two PGs, versican (*VCAN*) and decorin (*DCN*), was upregulated in pancreatic cancer (Skandalis et al., 2006). The *VCAN* expression level in pancreatic neuroendocrine tumor tissues was found to be higher than in normal pancreatic tissues (Gao et al., 2020). In this study, the RNA-seq data indicated that the expression levels of both *CHST3* and *CHST15* encoding chondroitin 6-*O*-sulfotransferase and GalNAc-4-*O*-sulfate 6-*O*-sulfotransferase in tumors were increased 4.0 and 3.5 times, respectively, in pancreatic tumors compared with that in normal tissues (Supplementary Figure 3 and Supplementary Table 4). The expression levels of *VCAN* and *DCN* were increased 4.8 and 1.5 times, respectively (Supplementary Table 4). Besides, it has been reported that *endocan* (*ESM1*) expression in pancreatic neuroendocrine tumor correlates with poor clinical outcomes (Lin et al., 2017). The overexpression of *lumican* (*LUM*) and *biglycan* (*BGN*) has been reported in pancreatic cancers (Weber et al., 2001; Ishiwata et al., 2007). A high *syndecan 1* (*SDC1*) mRNA level tended to increase the mortality rate due to pancreatic cancer (Wu et al., 2020). In our analysis, expression of all PGs except for *neurocan* (*NCAN*), which showed almost no expression, were increased in pancreatic carcinoma (Supplementary Figure 3F). Particularly, *VCAN*, *ESM1*, *LUM*, *BGN*, and *SDC1* were the five most upregulated PGs. Similar to brain tumors, genes encoding transporters and regulators related to GAG biosynthesis were also upregulated in pancreatic tumors (Supplementary Figure 3G and Supplementary Tables 4, 5).

**FIGURE 4:**
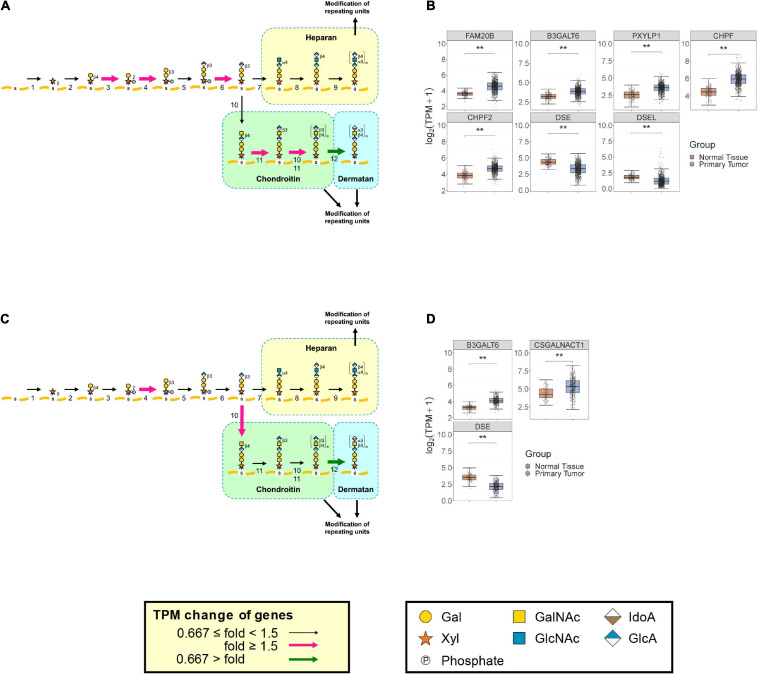
Comparison of expression of GAG biosynthetic enzymes between breast and prostate primary tumors and normal tissues. Expression changes of genes responsible for biosynthetic pathways for GAG backbones between primary breast tumors (*N* = 1092) and normal tissues (*N* = 179) **(A)** or primary prostate tumors (*N* = 495) and normal tissues (*N* = 100) **(C)** were visualized using GlycoMaple, as described in Figure 3. Fold changes of gene expression whose median TPM + 1 value was increased by >1.5 and decreased by <0.667 are shown as pink and green arrows, respectively. GAG core biosynthesis genes that were significantly changed among tumor and normal tissues (Wilcoxon matched-pairs signed rank test; **P < 0.0001) in breast **(B)** and prostate **(D)** are shown as boxplots. The numbers of each step are referred to in the legend of Figure 3.

In breast cancer, the DS content has been reported to decrease significantly, whereas the CS content increased in the central area of breast carcinoma tissues compared with fibroadenoma (Olsen et al., 1988). A similar change in GAG levels was observed in prostate cancer, where the DS level decreased and the CS level increased, when compared with normal tissue (De Klerk et al., 1984). GlycoMaple data including expression levels of biosynthetic enzymes for GAGs correlate well with these trends including the amounts of GAGs (Figure 4). In primary breast tumors, there is reduced expression of *DSE* and *DSEL*, which are the genes encoding DS epimerase converting GlcA to IdoA, to generate dermatan from the chondroitin precursor chain (Figures 4A,B). In addition, *CHPF* and *CHPF2*, whose products are required for the polymerization of chondroitin, were upregulated in breast cancer tissues (Figures 4A,B). Consistent with this result, down- and up-regulations of *DSE* and *CHPF*, respectively, were also observed in ductal carcinoma *in situ* compared with non-malignant breast tissue (Potapenko et al., 2015). It has also been demonstrated that among PG and GAG-related genes, *ACAN*, *VCAN*, *XYLT2*, *B3GALT6*, *CHSY1*, *CHPF*, *CHST11*, and *CHST15* were upregulated in malignant breast cancer tissue, while *CHST3* was downregulated (Potapenko et al., 2010). These findings were consistent with our data: expression levels of *ACAN*, *VCAN*, *B3GALT6*, *CHPF*, *CHST11*, and *CHST15* were increased more than 1.5 times in breast tumors, whereas expression of *CHST3* was 3.4-times decreased (Figure 4B, Supplementary Figure 4A, and Supplementary Table 4). In terms of PGs, it has been reported that *ESM1* was overexpressed in triple-negative breast cancer cell lines as well as in patient tissues, which is correlated with a poor prognosis (Fernandez-Vega et al., 2013). *SDC1* expression was activated, when compared with the very low level of expression in normal breast tissue, while expression of *DCN* decreased two—five-fold (Eshchenko et al., 2007). *BGN* was upregulated in human breast cancers, particularly in the tumor stroma compartment, compared with normal mammary glands (Cong et al., 2021). *VCAN* mRNA levels were upregulated in breast cancer tissues (Takahashi et al., 2012). In contrast, normal breast tissues exhibited high expression levels of *GPC3*, while the expression was reduced in tumors (Guereno et al., 2020). Despite the fact that *HSPG2* expression was correlated with poor patient survival and is considered as a therapeutic target in triple-negative breast cancer (Kalscheuer et al., 2019), the expression levels were two-fold higher in normal breast compared with breast cancer tissues (Jansson et al., 2020). Our analysis also showed changes in expression of those genes: upregulation of *SDC1*, *ESM1*, *VCAN*, and *BGN*, and downregulation of *GPC3*, *HSPG2*, and *DCN* in breast cancer tissues (Supplementary Figure 4A and Supplementary Tables 4, 5).

In primary prostate tumors, *DSE* expression was decreased to less than half (Figures 4C,D). In addition, a greater than two-fold increase was found in *CSGALNACT1*, whose product initiates the biosynthesis of the disaccharide region of the chondroitin chain (Figures 4C,D). These results are consistent with the findings of decreased DS and increased CS in pancreatic cancerous tissues (De Klerk et al., 1984), suggesting that GlycoMaple is a powerful tool for estimating changes of GAGs in diseased tissues. In terms of PGs, it has been reported that the expression of *fibromodulin* (*FMOD*) in malignant prostate tissues is significantly upregulated compared with that in benign tissues (Bettin et al., 2016). A significant decrease in tissue *DCN* expression is associated with tumor progression and metastasis in certain types of cancer including prostate cancer (Rezaie et al., 2020). The *GPC2* mRNA expression level was utilized to predict survival associated with prostate cancer (Xu et al., 2018). In our analysis, upregulations of *neuroglycan C* (*CSPG5*), *bamacan* (*SMC3*), and *collagen type IX alpha2* (*COL9A2*) and downregulations of *GPC1*, *GPC2*, *HSPG2*, *CSPG4*, and *DCN* were detected (Supplementary Figure 4B and Supplementary Table 5).

Finally, we estimated GAG changes in the adrenal and thyroid glands during tumorization. GlycoMaple analysis predicted decreased levels of CS and DS, because of the downregulation of *CSGALNACT1* and *DSE* in both the adrenal gland and thyroid gland tumors compared with that in normal tissues (Supplementary Figure 5). To date, there has been no report that GAG levels are changed in those tissues during tumorization. Therefore, it is worth examining the amounts of CS and DS in tumor tissues.

## Concluding Remarks

In this study, we analyzed the expression levels of GAG biosynthetic enzymes as well as PGs in various normal and tumor tissues. GAG biosynthetic levels differed, depending upon the tissue functions and requirements. Analyses of the amount and sulfation pattern of GAGs are not easy to perform and require relatively abundant starting materials. The estimation of glycan structures based on gene expression data could be performed easily from small amounts of samples, and is useful to discover properties of cells of interest and obtain clues to structural changes among cell types. We applied GlycoMaple analysis to visualize the expression of genes involved in GAG biosynthesis and PG levels and to estimate glycan changes based on gene expression. Comprehensive analysis using gene expression levels in human tissues revealed new findings showing that expression of some genes required for GAG biosynthesis are regulated in a similar manner. For example, expression of genes required for the biosynthesis of GAG backbones including CS, DS, HS, and HA may be similarly regulated. GlycoMaple estimation showed that GAG biosynthetic patterns and core proteins of PGs were markedly changed during tumor progression in some tissues, which correlated with previous research. The process of CS formation would be upregulated during the formation of many tumor types (Berto et al., 2001; Sakko et al., 2008; Svensson et al., 2011). However, there are several limitations in GlycoMaple analysis (Huang et al., 2021). First, estimations of GAG changes in tumors using GlycoMaple, are just predictions based on expression changes of genes related to GAG biosynthesis. Second, the estimation of glycans from GlycoMaple is not quantitative. Therefore, after identifying potential target pathways from these estimations, it is essential to validate GAG as well as PG levels using biochemical analyses. Nonetheless, GlycoMaple could provide a ‘bird’s eye view’ of glycosylation pathways in cells/tissues of interest and clues for focusing on altered glycosylation pathways between diseased and normal tissues. Changes in glycosylation patterns during tumor progression were widely observed. In the future, GlycoMaple analysis could contribute to the development of biomarkers and clinical diagnostics using transcriptional data from clinical patient samples.

## Data Availability Statement

The original contributions generated for this study are included in the article/Supplementary Material, further inquiries can be directed to the corresponding author.

## Author Contributions

MF and Y-FH conceptualized and designed the study and wrote a draft of the manuscript. Y-FH conducted analyses and validated the results. SM strengthened the background of GAG. All authors checked and edited the manuscript.

## Conflict of Interest

The authors declare that the research was conducted in the absence of any commercial or financial relationships that could be construed as a potential conflict of interest.

## Publisher’s Note

All claims expressed in this article are solely those of the authors and do not necessarily represent those of their affiliated organizations, or those of the publisher, the editors and the reviewers. Any product that may be evaluated in this article, or claim that may be made by its manufacturer, is not guaranteed or endorsed by the publisher.

## Footnotes

1
https://www.proteinatlas.org/download/rna_tissue_hpa.tsv.zip

2
https://xenabrowser.net/
