# Supplementary material for: Novel Insight Into Glycosaminoglycan Biosynthesis Based on Gene Expression Profiles
Source: Front Cell Dev Biol. 2021 Sep 6;9:709018. doi: 10.3389/fcell.2021.709018 (PMC8450405; doi:10.3389/fcell.2021.709018)
Supplement: Supplementary file 1 [file Data_Sheet_1.PDF]

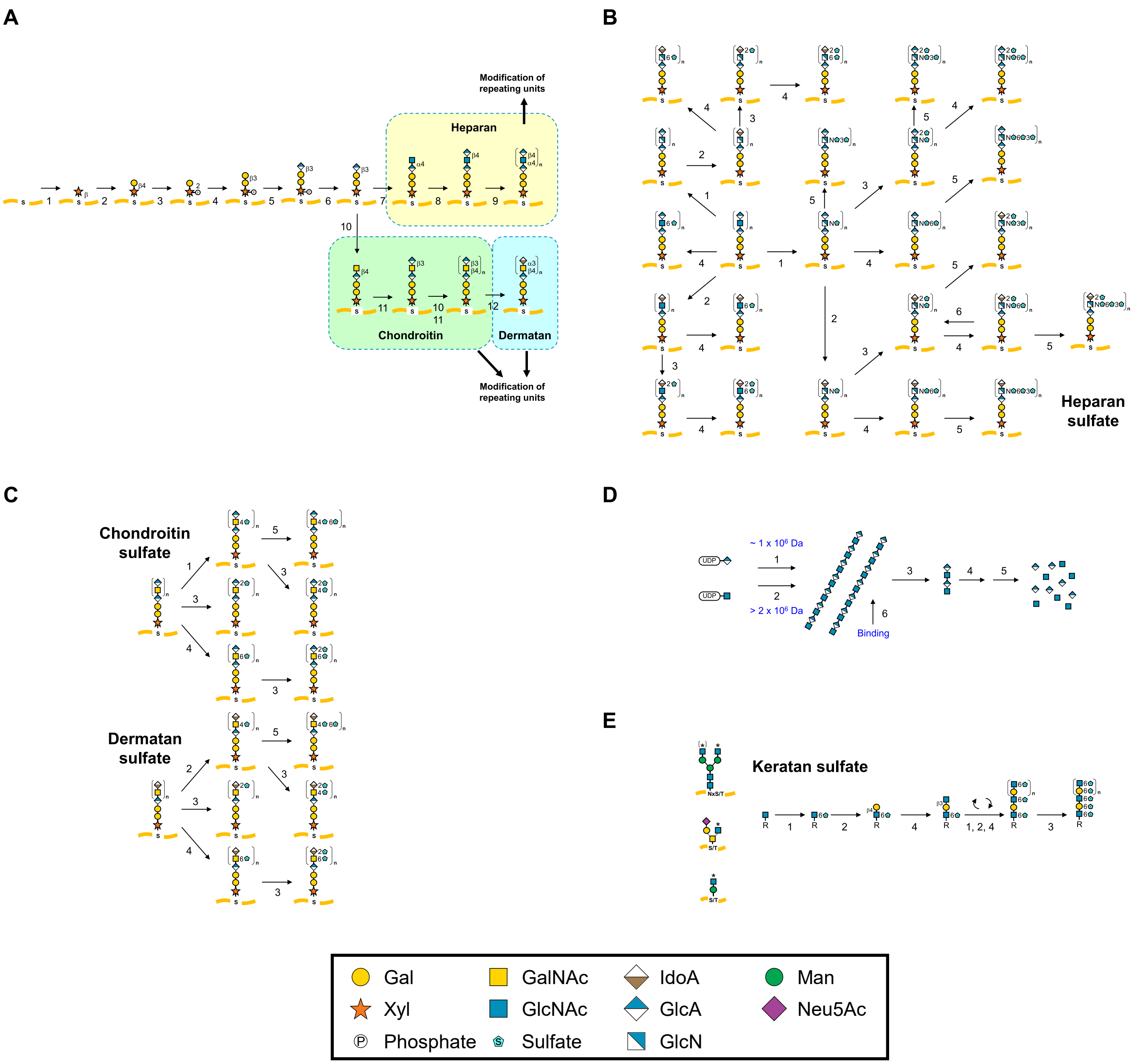

Figure S1. Pathways for glycosaminoglycan biosynthesis.
Glycosaminoglycan (GAG) biosynthetic pathways are shown including biosynthesis of GAG backbones (A), biosynthesis of heparan sulfate (HS) (B), biosynthesis of chondroitin sulfate (CS) and dermatan sulfate (DS) (C), biosynthesis and catabolism of hyaluronan (HA) (D), and biosynthesis of keratan sulfate (KS) (E). The numbers of each step indicate the enzymes responsible for biosynthesis of GAGs (refer to Table S1).

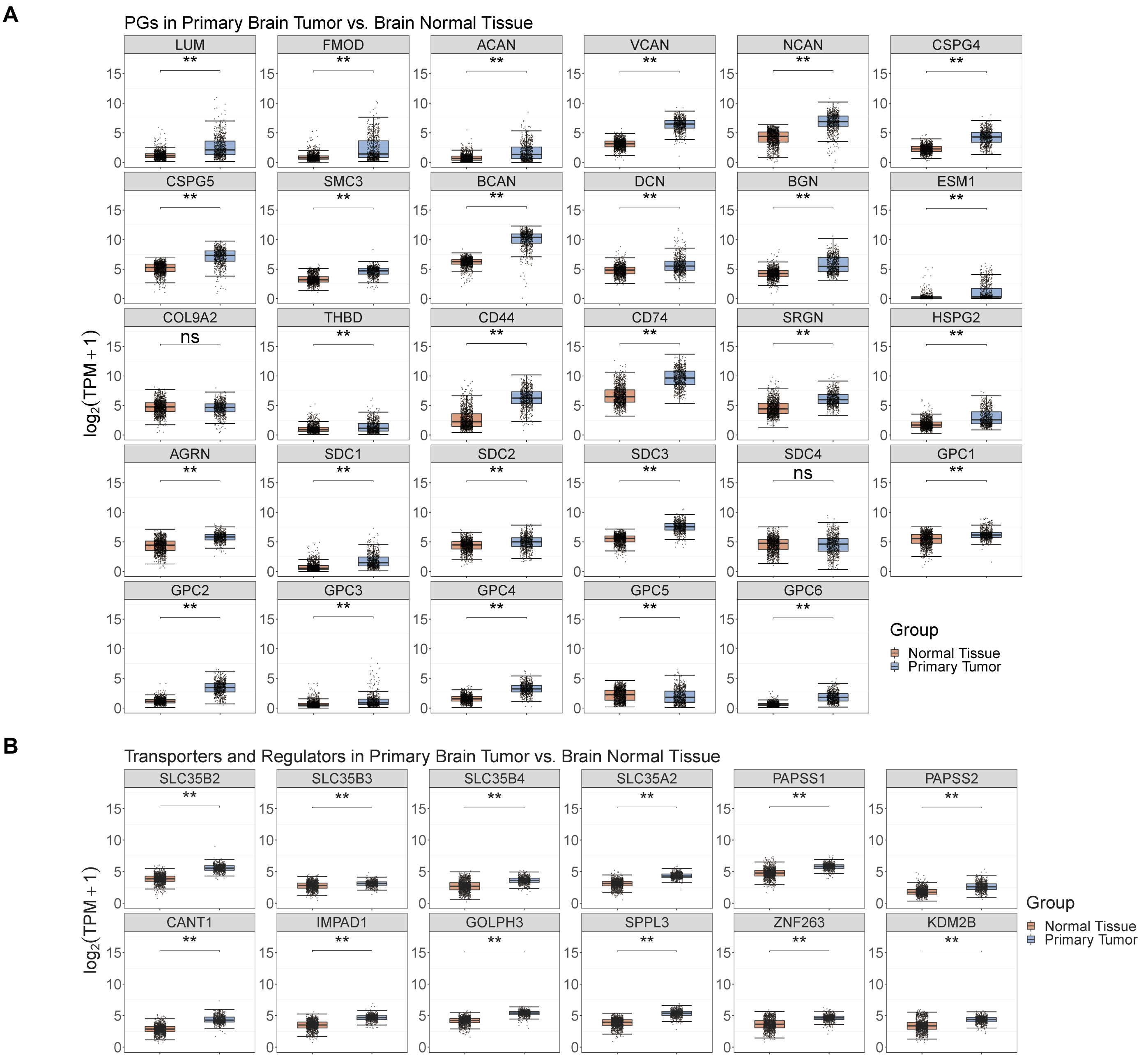

Figure S2. Comparison of expression levels of genes encoding core proteins of proteoglycans, nucleotide sugar transporters, 3'-phosphoadenosine 5'-phosphosulfate synthase, calcium-activated nucleotidase, 3'-phosphoadenosine 5'-phosphate 3'-phosphatase, and regulators between primary brain tumors and normal tissues.

Expression of genes encoding core proteins of proteoglycans (PGs) (A), nucleotide sugar transporters, 3'-phosphoadenosine 5'-phosphosulfate (PAPS) synthase, calcium-activated nucleotidase, 3'-phosphoadenosine 5'-phosphate 3'-phosphatase, and regulators (B) were compared between primary brain tumors (N = 662) and normal tissues (N = 1146). Wilcoxon matched-pairs signed rank test was used to compare expression between tumor and normal tissues. Differences with $p < 0.0001$ (**) and non-significant (ns) are indicated. Gene expressions of 27 PGs, except *COL9A2* and *SDC4*, were upregulated in primary brain tumors.

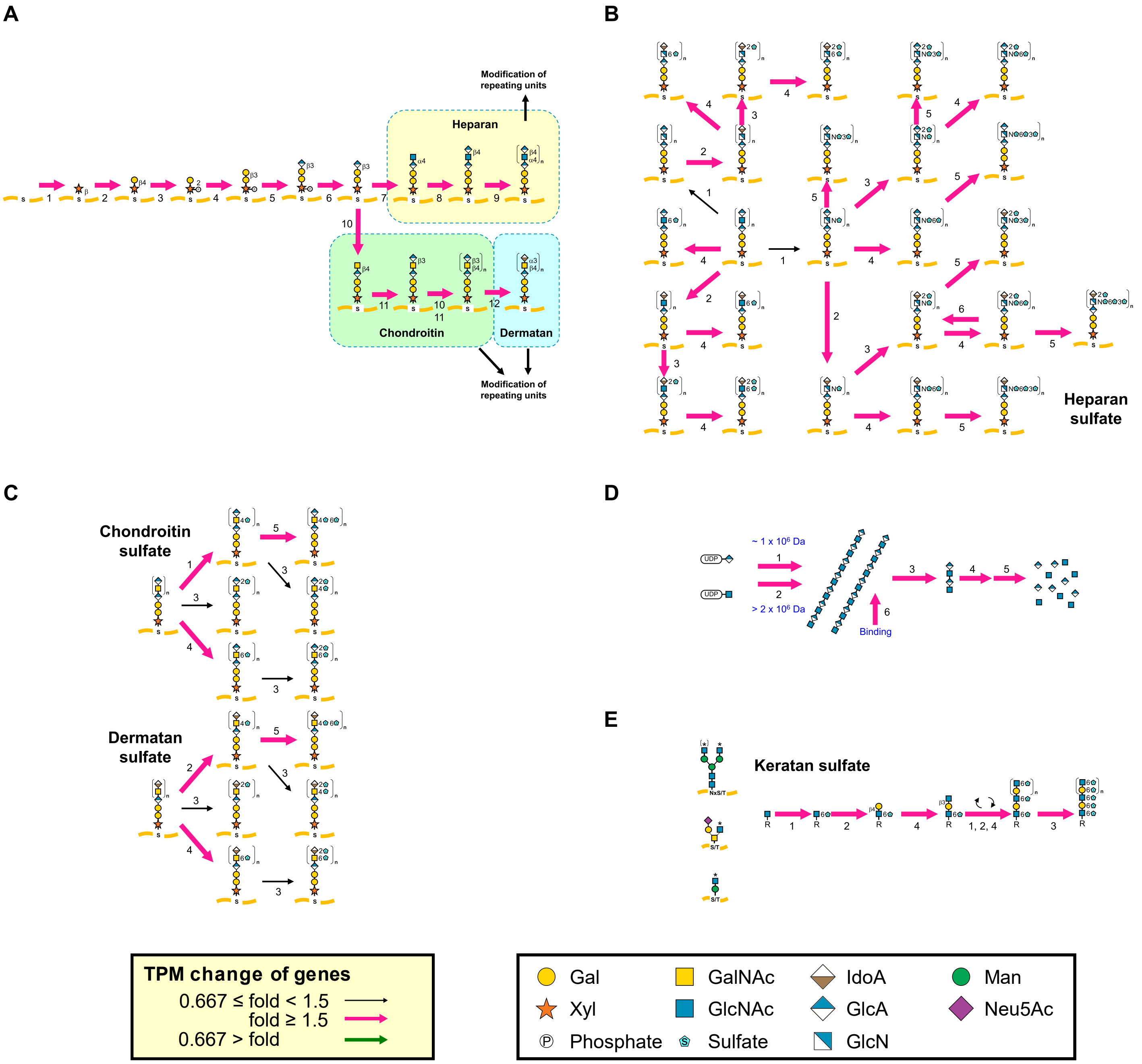
A
Modification of repeating units
Heparan
1
2
3
4
5
6
7
8
9
10
Chondroitin
Dermatan
11
12
Modification of repeating units
B
Heparan sulfate
C
Chondroitin sulfate
Dermatan sulfate
D
~ 1 x 10⁶ Da
> 2 x 10⁶ Da
UDP
Binding
E
Keratan sulfate
NxS/T
S/T
1, 2, 4
TPM change of genes
0.667 ≤ fold < 1.5
fold ≥ 1.5
0.667 > fold
Gal
Xyl
Phosphate
GalNAc
GlcNAc
Sulfate
IdoA
GlcA
GlcN
Man
Neu5Ac

F

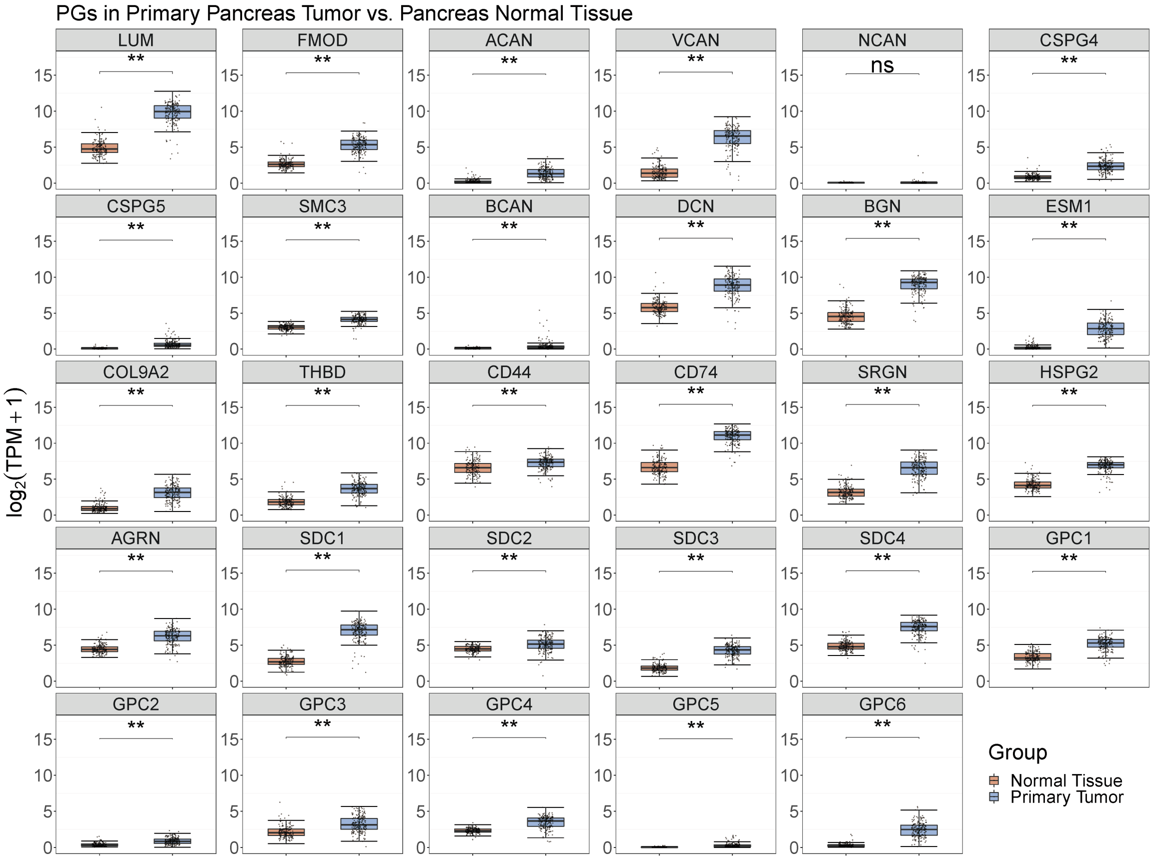

G

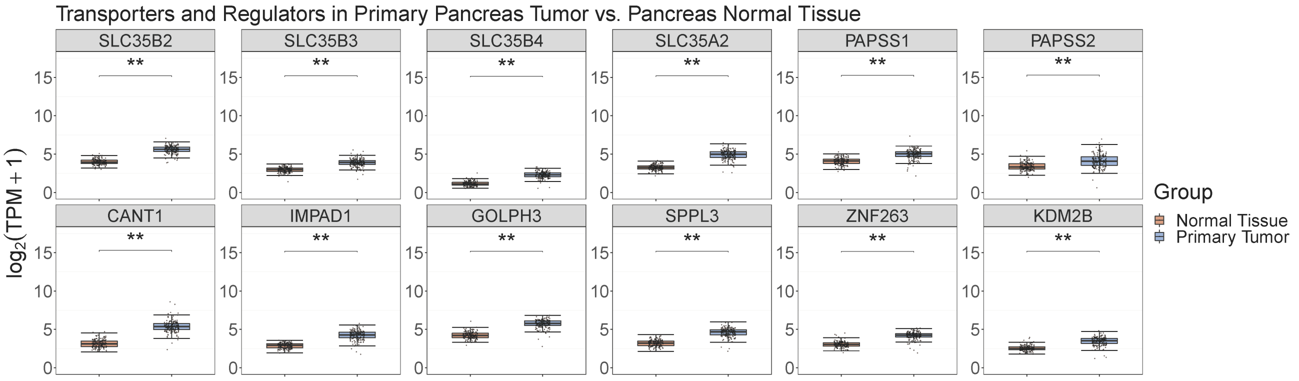

Figure S3. Comparison of expression levels of genes encoding GAG biosynthetic enzymes, core proteins of proteoglycans, nucleotide sugar transporters, PAPS synthase, calcium-activated nucleotidase, 3’-phosphoadenosine 5’-phosphate 3’-phosphatase, and regulators between pancreas primary tumor and normal tissues.

Comparisons of GAG biosynthetic pathways are shown as pink arrows, including: A, biosynthesis of GAG backbones; B, sulfation of HS; C, sulfation of CS and DS; D, biosynthesis and catabolism of HA; and E, biosynthesis of KS between primary pancreas tumors (N = 178) and normal tissues (N = 165), visualized using GlycoMaple. Fold changes of gene expression whose median TPM+1 value was increased by > 1.5. Expression of genes encoding core proteins of PGs (F), nucleotide sugar transporters, 3'-phosphoadenosine 5'-phosphosulfate (PAPS) synthase, calcium-activated nucleotidase, 3’-phosphoadenosine 5’-phosphate 3’-phosphatase, and regulators (G) were compared between primary pancreas tumors (N = 178) and normal tissues (N = 165). Wilcoxon matched-pairs signed rank test was used to compare expression between tumor and normal tissues. Differences with $p < 0.0001$ (**) and non-significant (ns) are indicated. The numbers of each step indicate the enzymes responsible for biosynthesis as well as catabolism of GAGs (refer to Table S1).

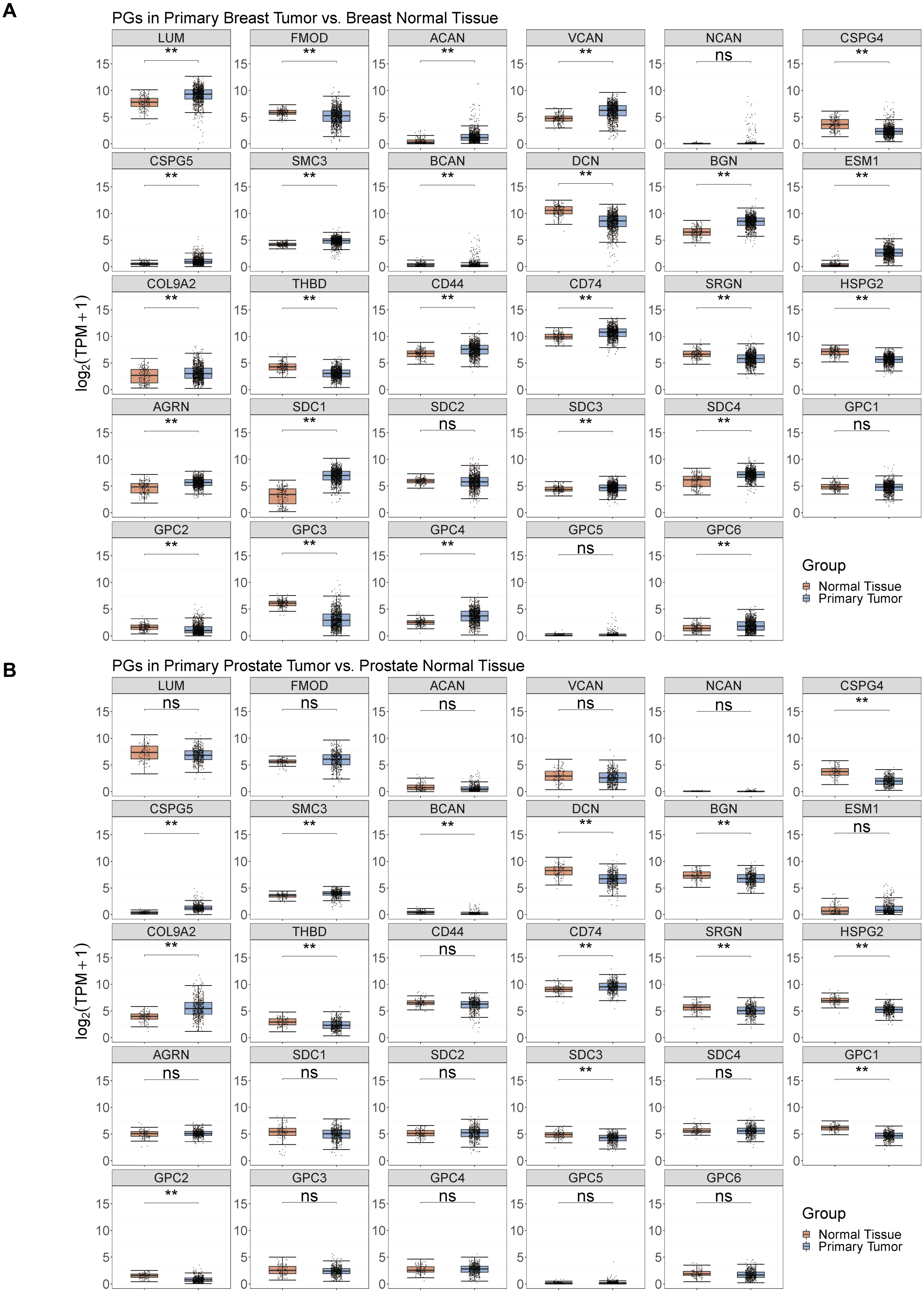

Figure S4. Comparison of expression levels in core proteins PGs between primary tumor and normal tissues from the breast and prostate.

Expression of genes encoding core proteins of PGs were compared between primary breast tumors (*N* = 1092) and normal tissues (*N* = 179) (A) or between primary prostate tumors (*N* = 495) and normal tissues (*N* = 100) (B). Wilcoxon matched-pairs signed rank test was used to compare expression between tumor and normal tissues. Differences with $p < 0.0001$ (**) and non-significant (ns) are indicated.

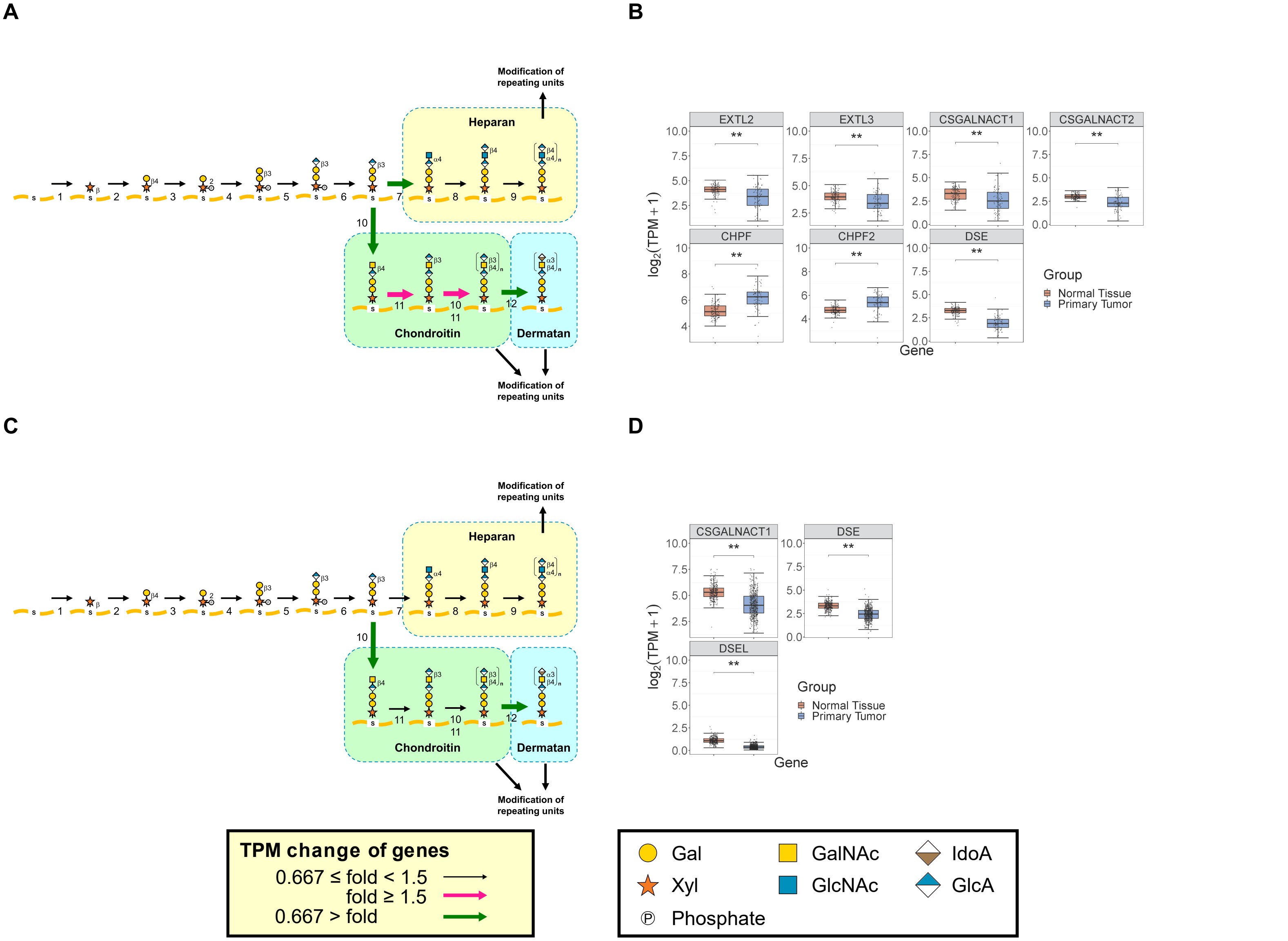

Figure S5. Comparison of expression levels of GAG biosynthetic genes in adrenal and thyroid glands. Expression changes of genes responsible for GAG backbones between primary adrenal gland tumors (N = 77) and normal tissues (N = 127) (A) or between primary thyroid gland tumors (N = 504) and normal tissues (N = 278) (C) visualized using GlycoMaple. Fold changes of gene expression whose median TPM+1 value was increased by > 1.5 and decreased by < 0.667 are shown as pink and green arrows, respectively. Genes that are significantly changed among tumor and normal tissues in GAG core biosynthesis in the adrenal gland (B) or thyroid gland (D) are shown as boxplots. Wilcoxon matched-pairs signed rank test was used to compare expression between tumor and normal tissues. Differences with $p < 0.0001$ (**) and non-significant (ns) are indicated. The numbers of each step indicate the enzymes responsible for biosynthesis as well as catabolism of GAGs (refer to Table S1).
